# Supplementary material for: Identification of potent and selective N-myristoyltransferase inhibitors of Plasmodium vivax liver stage hypnozoites and schizonts
Source: Nat Commun. 2023 Sep 5;14:5408. doi: 10.1038/s41467-023-41119-7 (PMC10480161; doi:10.1038/s41467-023-41119-7)
Supplement: Supplementary file 3 — Reporting Summary [file 41467_2023_41119_MOESM3_ESM.pdf]

nature portfolio

Corresponding author(s): Morten Grötli/Alexisi Kaushansky

Last updated by author(s): Jul 26, 2023

Reporting Summary

Nature Portfolio wishes to improve the reproducibility of the work that we publish. This form provides structure for consistency and transparency in reporting. For further information on Nature Portfolio policies, see our Editorial Policies and the Editorial Policy Checklist.

Statistics

For all statistical analyses, confirm that the following items are present in the figure legend, table legend, main text, or Methods section.

n/a	Confirmed	
☐	☒	The exact sample size (*n*) for each experimental group/condition, given as a discrete number and unit of measurement
☐	☒	A statement on whether measurements were taken from distinct samples or whether the same sample was measured repeatedly
☐	☒	The statistical test(s) used AND whether they are one- or two-sided<br>*Only common tests should be described solely by name; describe more complex techniques in the Methods section.*
☐	☒	A description of all covariates tested
☐	☒	A description of any assumptions or corrections, such as tests of normality and adjustment for multiple comparisons
☐	☒	A full description of the statistical parameters including central tendency (e.g. means) or other basic estimates (e.g. regression coefficient) AND variation (e.g. standard deviation) or associated estimates of uncertainty (e.g. confidence intervals)
☐	☒	For null hypothesis testing, the test statistic (e.g. *F*, *t*, *r*) with confidence intervals, effect sizes, degrees of freedom and *P* value noted<br>*Give P values as exact values whenever suitable.*
☐	☒	For Bayesian analysis, information on the choice of priors and Markov chain Monte Carlo settings
☐	☒	For hierarchical and complex designs, identification of the appropriate level for tests and full reporting of outcomes
☐	☒	Estimates of effect sizes (e.g. Cohen's *d*, Pearson's *r*), indicating how they were calculated

Our web collection on statistics for biologists contains articles on many of the points above.

Software and code

Policy information about availability of computer code

Data collection	No software was used.
Data analysis	IMARIS, Oxford instruments for Image analysis; GraphPad for data analysis

For manuscripts utilizing custom algorithms or software that are central to the research but not yet described in published literature, software must be made available to editors and reviewers. We strongly encourage code deposition in a community repository (e.g. GitHub). See the Nature Portfolio guidelines for submitting code & software for further information.

Data

Policy information about availability of data

All manuscripts must include a data availability statement. This statement should provide the following information, where applicable:

- Accession codes, unique identifiers, or web links for publicly available datasets
- A description of any restrictions on data availability
- For clinical datasets or third party data, please ensure that the statement adheres to our policy

The raw X-ray diffraction images generated in this study have been deposited in the Integrated Resource for Reproducibility in Macromolecular Crystallography under accession code 8FBQ at https://proteindiffraction.org62. Protein expression plasmids of PvNMT, HsNMT1 and HsNMT2 are available at https://www.ssgcid.org/available-materials/ssgcid-proteins/ through a material transfer agreement (MTA). Source data are provided with this paper. The accession codes of all PDB coordinate files referenced in this study, including that of PvNMT-myrCoA-12b (8FBQ), are listed below together with the associated web addresses at the

wwPDB:

2YNC https://www.wwpdb.org/pdb?id=pdb_2YNC

2YND https://www.wwpdb.org/pdb?id=pdb_2YND

2YNE https://www.wwpdb.org/pdb?id=pdb_2YNE

3IU1 https://www.wwpdb.org/pdb?id=pdb_3IU1

3IU2 https://www.wwpdb.org/pdb?id=pdb_3IU2

3IWE https://www.wwpdb.org/pdb?id=pdb_3IWE

3JTK https://www.wwpdb.org/pdb?id=pdb_3JTK

4A95 https://www.wwpdb.org/pdb?id=pdb_4A95

4B10 https://www.wwpdb.org/pdb?id=pdb_4B10

4B11 https://www.wwpdb.org/pdb?id=pdb_4B11

4B12 https://www.wwpdb.org/pdb?id=pdb_4B12

4B13 https://www.wwpdb.org/pdb?id=pdb_4B13

4B14 https://www.wwpdb.org/pdb?id=pdb_4B14

4BBH https://www.wwpdb.org/pdb?id=pdb_4BBH

4C2Y https://www.wwpdb.org/pdb?id=pdb_4C2Y

4C2Z https://www.wwpdb.org/pdb?id=pdb_4C2Z

4C68 https://www.wwpdb.org/pdb?id=pdb_4C68

4CAE https://www.wwpdb.org/pdb?id=pdb_4CAE

4CAF https://www.wwpdb.org/pdb?id=pdb_4CAF

4UFV https://www.wwpdb.org/pdb?id=pdb_4UFV

4UFW https://www.wwpdb.org/pdb?id=pdb_4UFW

4UFX https://www.wwpdb.org/pdb?id=pdb_4UFX

5G1Z https://www.wwpdb.org/pdb?id=pdb_5G1Z

5G22 https://www.wwpdb.org/pdb?id=pdb_5G22

5MU6 https://www.wwpdb.org/pdb?id=pdb_5MU6

5O48 https://www.wwpdb.org/pdb?id=pdb_5O48

5O4V https://www.wwpdb.org/pdb?id=pdb_5O4V

5O6H https://www.wwpdb.org/pdb?id=pdb_5O6H

5O6J https://www.wwpdb.org/pdb?id=pdb_5O6J

5NPQ https://www.wwpdb.org/pdb?id=pdb_5NPQ

5O9S https://www.wwpdb.org/pdb?id=pdb_5O9S

5O9T https://www.wwpdb.org/pdb?id=pdb_5O9T

5O9U https://www.wwpdb.org/pdb?id=pdb_5O9U

5O9V https://www.wwpdb.org/pdb?id=pdb_5O9V

5UUT https://www.wwpdb.org/pdb?id=pdb_5UUT

5V0W https://www.wwpdb.org/pdb?id=pdb_5V0W

5V0X https://www.wwpdb.org/pdb?id=pdb_5V0X

6B1L https://www.wwpdb.org/pdb?id=pdb_6B1L

6FZ2 https://www.wwpdb.org/pdb?id=pdb_6FZ2

6FZ3 https://www.wwpdb.org/pdb?id=pdb_6FZ3

6FZ5 https://www.wwpdb.org/pdb?id=pdb_6FZ5

6MB1 https://www.wwpdb.org/pdb?id=pdb_6MB1

6NXG https://www.wwpdb.org/pdb?id=pdb_6NXG

6PAV https://www.wwpdb.org/pdb?id=pdb_6PAV

6EHJ https://www.wwpdb.org/pdb?id=pdb_6EHJ

6QRM https://www.wwpdb.org/pdb?id=pdb_6QRM

6SJZ https://www.wwpdb.org/pdb?id=pdb_6SJZ

6SK2 https://www.wwpdb.org/pdb?id=pdb_6SK2

6TW5 https://www.wwpdb.org/pdb?id=pdb_6TW5

6TW6 https://www.wwpdb.org/pdb?id=pdb_6TW6

7RK3 https://www.wwpdb.org/pdb?id=pdb_7RK3

8FBQ https://www.wwpdb.org/pdb?id=pdb_8FBQ

Research involving human participants, their data, or biological material

Policy information about studies with human participants or human data. See also policy information about sex, gender (identity/presentation), and sexual orientation and race, ethnicity and racism.

Reporting on sex and gender	The volunteers who donated blood to obtained P. vivax-infected mosquitoes were obtained as part of a separate study, and thus are de-identified as part of this study. For this reason, we are unable to report on the volunteer population.
Reporting on race, ethnicity, or other socially relevant groupings	See above.
Population characteristics	See above.
Recruitment	See above.
Ethics oversight	See above.

Note that full information on the approval of the study protocol must also be provided in the manuscript.

Field-specific reporting

Please select the one below that is the best fit for your research. If you are not sure, read the appropriate sections before making your selection.

☒ Life sciences ☐ Behavioural & social sciences ☐ Ecological, evolutionary & environmental sciences

For a reference copy of the document with all sections, see nature.com/documents/nr-reporting-summary-flat.pdf

Life sciences study design

All studies must disclose on these points even when the disclosure is negative.

Sample size	Sample size of three technical replicates per biological replicate and three biological replicates was used for statistical analysis.
Data exclusions	No data were excluded.
Replication	Replication was successful. P. vivax experiments could only be preformed once per isolate as each comes from a unique individual.
Randomization	Location of compounds on the plate was randomized
Blinding	Quantification was preformed with blinding to treatment.

Reporting for specific materials, systems and methods

We require information from authors about some types of materials, experimental systems and methods used in many studies. Here, indicate whether each material, system or method listed is relevant to your study. If you are not sure if a list item applies to your research, read the appropriate section before selecting a response.

Materials & experimental systems

n/a	Involved in the study
☐	☒ Antibodies
☐	☒ Eukaryotic cell lines
☒	☐ Palaeontology and archaeology
☒	☐ Animals and other organisms
☒	☐ Clinical data
☒	☐ Dual use research of concern
☒	☐ Plants

Methods

n/a	Involved in the study
☒	☐ ChIP-seq
☒	☐ Flow cytometry
☒	☐ MRI-based neuroimaging

Antibodies

Antibodies used	P. vivax UIS4-targeting antibody provided by Sather lab. For antibody dilutions PvUIS4 was diluted 1:250 and secondary antibody was anti-mouse AlexaFluor-594 diluted 1:1000. DAPI was diluted 1:2000.
Validation	Validated in Schafer et al 2018, Malaria Journal.

Eukaryotic cell lines

Policy information about cell lines and Sex and Gender in Research

Cell line source(s)	Primary human hepatocytes from Bio IVT
Authentication	Lot autenticated by Both et al. 2028, Nat Commun.
Mycoplasma contamination	Not tested for mycoplasma
Commonly misidentified lines (See ICLAC register)	n/a
